# Supplementary material for: An Experimentally Verified Mechanistic Model for Predicting Quorum Sensing‐Based Switches
Source: Microb Biotechnol. 2026 Jul 13;19(7):e70408. doi: 10.1111/1751-7915.70408 (PMC13365800; doi:10.1111/1751-7915.70408)
Supplement: Supplementary file 1 — Figure S1: Overview of the growth and fluorescence output of the 15 strains from the EsaI/EsaR library (part 1). The library consists of strains with different expression levels of the synthase, regulated by a promoter library (first part of the subplot titles), and different ribosome binding sites for the transcription factor EsaR (second part of the subplot titles). P esaR and P esaS activity are quantified by red fluorescent protein mKate2 and green fluorescent sfGFP production, respectively. The strain constitutively expresses EsaR, which, in the absence of its autoinducer, binds the promoter region leading to an activation of P esaR and repression of P esaR. Additionally, the respective synthase EsaI is constitutively expressed. Fluorescent values are normalized for cell growth determined by optical density at 600 nm (OD600). Error bars represent the standard error for three biological replicates. Figure S2: Overview of the growth and fluorescence output of the 15 strains from the EsaI/EsaR library (part 2). The library consists of strains with different expression levels of the synthase, regulated by a promoter library (first part of the subplot titles), and different ribosome binding sites for the transcription factor EsaR (second part of the subplot titles). P esaR and P esaS activity are quantified by red fluorescent protein mKate2 and green fluorescent sfGFP production, respectively. The strain constitutively expresses EsaR, which, in the absence of its autoinducer, binds the promoter region leading to an activation of P esaR and repression of P esaR. Additionally, the respective synthase EsaI is constitutively expressed. Fluorescent values are normalized for cell growth determined by optical density at 600 nm (OD600). Error bars represent the standard error for three biological replicates. Figure S3: Growth curves of each of the three biological replicates of the 15 strains of the EsaI/EsaR library created for model fitting (part 1). The log‐transformed o [file MBT2-19-e70408-s001.pdf]

Appendix

An experimentally verified mechanistic model for predicting quorum sensing-based switches

Jasmine De Baets, Brecht De Paepe, Marjan De Mey

Centre for Synthetic Biology, Ghent university, 9000 Ghent, Belgium

Overview

Supplementary Text S1: Derivation of the equation of the AHL concentration.
Supplementary Figure S1: Overview of the fifteen strains of the EsaI/EsaR library (part 1).
Supplementary Figure S2: Overview of the fifteen strains of the EsaI/EsaR library (part 2).
Supplementary Figure S3: Overview of the fitted growth curves (part 1).
Supplementary Figure S4: Overview of the fitted growth curves (part 2).
Supplementary Figure S5: Results of the model fitted to the full quantifiable library (J23104 included).
Supplementary Figure S6: Results of the model fitted to final six library strains (J23104 excluded) with high parameter correlations.
Supplementary Figure S7: Traces of the walkers of the Monte Carlo Markov Chain analysis for the model with highly correlated parameters.
Supplementary Figure S8: Traces of the walkers of the Monte Carlo Markov Chain analysis for the model with reduced correlations between the parameters.
Supplementary Table S1: Overview of the fitted growth parameters.
Supplementary Table S2: Overview of the fitted (correlated) parameters to the six final strains.
Supplementary Table S3: Overview of the DNA-sequence of all regulatory parts used in this research.
Supplementary Table S4: Overview of the genes used in this research.
Supplementary Table S5: Overview of the primers used in this research.

S1 Derivation of the equation of the AHL concentration.

The dynamics of the total AHL concentration can be split up into different parts, corresponding to the production of the AHLs and their association and dissociation with EsaR:

$$\frac{d[AHL]}{dt} = \frac{d[AHL_{prod}]}{dt} - \frac{d[AHL_{ass}]}{dt} + \frac{d[AHL_{diss}]}{dt} \tag{1}$$

In this section, the focus lies on the derivation of the first part, namely the production of the AHL molecules. We consider diffusion not a rate-limiting step, making the intra- and extracellular AHL-concentration equal. The total number of AHL molecules produced and diffused can be written as:

$$TotalAHL_{prod} = [AHL_{prod}]V_{ext} \tag{2}$$

where V_{ext} corresponds to the available extracellular volume and can be calculated as

$$V_{ext} = V_{tot} - N_c(t)V_c \tag{3}$$

which subtracts the volume used by the increasing number of cells ($N_c(t)V_c$ with V_c the cell volume) from the total vessel volume (V_{tot}).
Since V_{ext} depends on time ($N_c(t)$), the time derivative can be expanded as follows:

$$\frac{d([AHL_{prod}]V_{ext})}{dt} = \frac{d[AHL_{prod}]}{dt}V_{ext} + [AHL_{prod}]\frac{dV_{ext}}{dt} \tag{4}$$

For simplicity, it can be assumed that changes in $N_c(t)V_c$ are slow, making $\frac{dV_{ext}}{dt} \approx 0$, thereby reducing Equation 4 to:

$$\frac{d([AHL_{prod}]V_{ext})}{dt} \approx \frac{d[AHL_{prod}]}{dt}V_{ext} \tag{5}$$

The total number of AHL is related to what is being produced by all the cells at a certain time point, as given in Equation 6, which can further be converted to the final equation for $\frac{d[AHL_{prod}]}{dt}$:

$$\begin{aligned}
\frac{d[AHL_{prod}]}{dt}V_{ext} &= [AHL_{prod}]_c N_c(t)V_c \\
&= v[EsaI]N_c(t)V_c \\
\frac{d[AHL_{prod}]}{dt} &= v[EsaI]N_c(t)\frac{V_c}{V_{ext}} \\
\frac{d[AHL_{prod}]}{dt} &= v[EsaI]N_c(t)\frac{V_c}{V_{tot} - N_c(t)V_c}
\end{aligned} \tag{6}$$

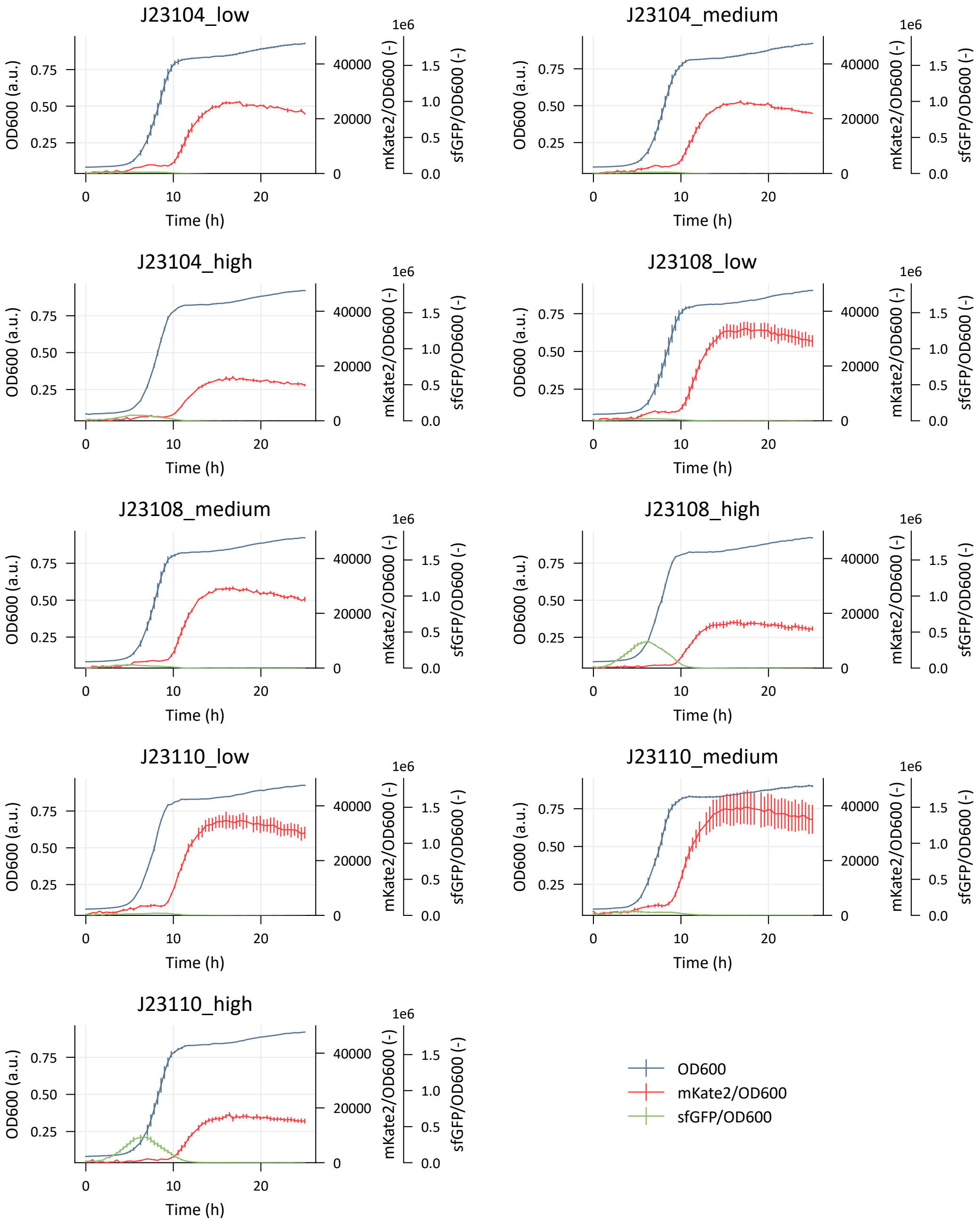

Figure S1: Overview of the growth and fluorescence output of the fifteen strains from the EsaI/EsaR library (part 1). The library consists of strains with different expression levels of the synthase, regulated by a promoter library (first part of the subplot titles), and different ribosome binding sites for the transcription factor EsaR (second part of the subplot titles). P_{esaR} and P_{esaS} activity are quantified by red fluorescent protein mKate2 and green fluorescent sfGFP production, respectively. The strain constitutively expresses EsaR, which, in the absence of its autoinducer, binds the promoter region leading to an activation of P_{esaR} and repression of P_{esaR}. Additionally, the respective synthase EsaI is constitutively expressed. Fluorescent values are normalized for cell growth determined by optical density at 600 nm (OD600). Error bars represent the standard error for three biological replicates.

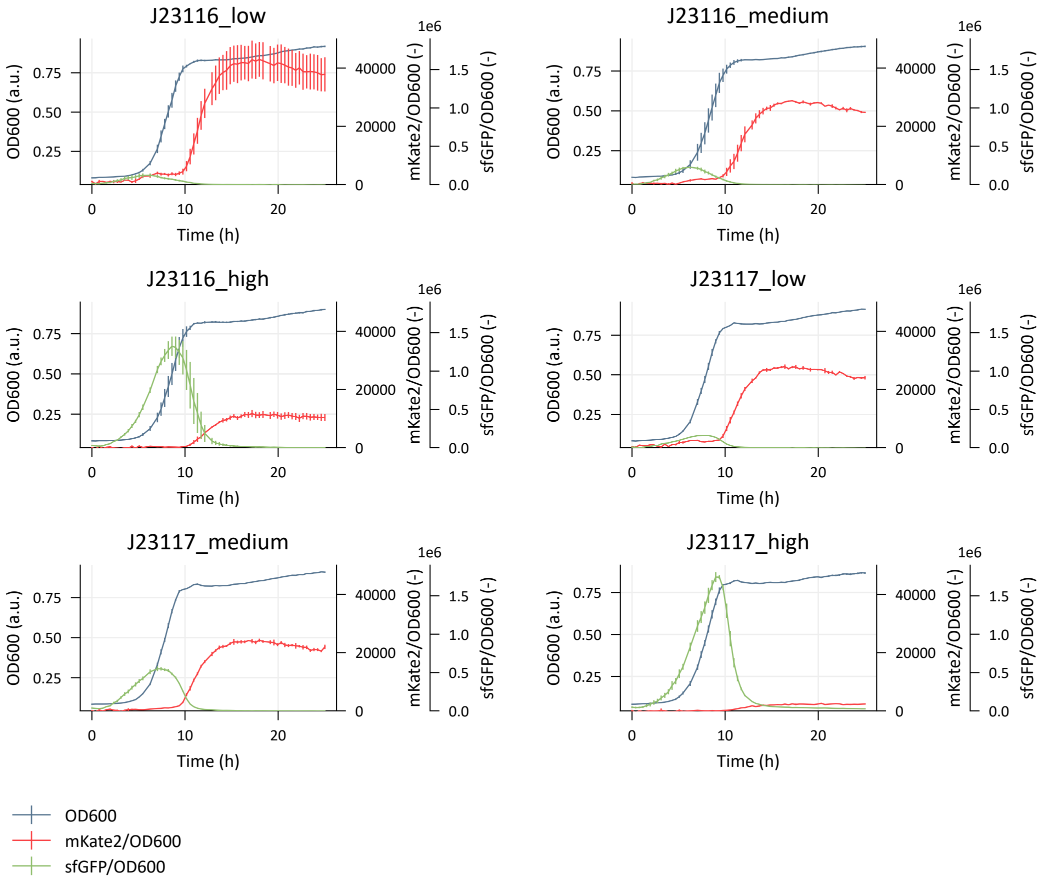

Figure S2: Overview of the growth and fluorescence output of the fifteen strains from the EsaI/EsaR library (part 2). The library consists of strains with different expression levels of the synthase, regulated by a promoter library (first part of the subplot titles), and different ribosome binding sites for the transcription factor EsaR (second part of the subplot titles). P_{esaR} and P_{esaS} activity are quantified by red fluorescent protein mKate2 and green fluorescent sfGFP production, respectively. The strain constitutively expresses EsaR, which, in the absence of its autoinducer, binds the promoter region leading to an activation of P_{esaR} and repression of P_{esaR}. Additionally, the respective synthase EsaI is constitutively expressed. Fluorescent values are normalized for cell growth determined by optical density at 600 nm (OD600). Error bars represent the standard error for three biological replicates.

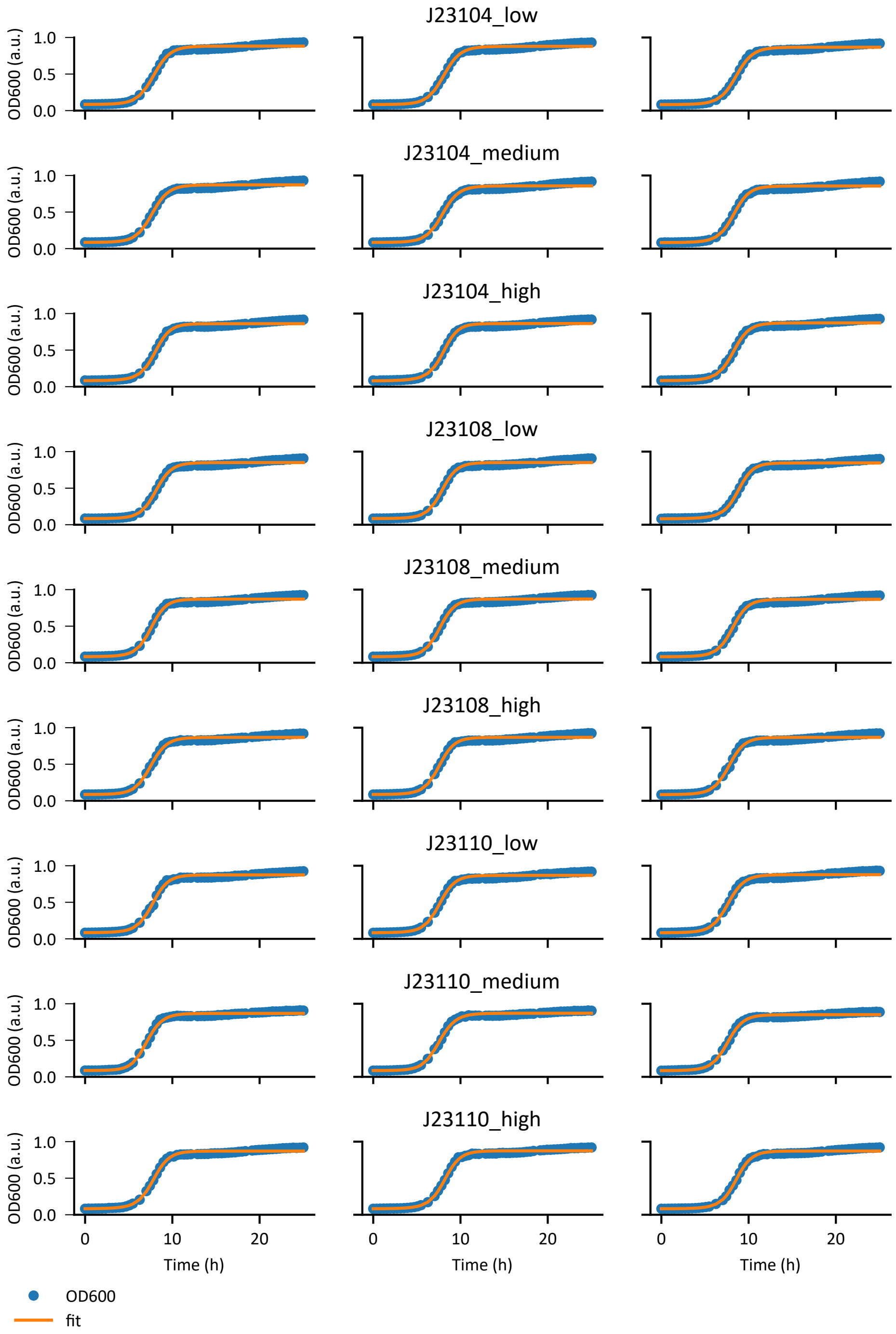

Figure S3: Growth curves of each of the three biological replicates of the fifteen strains of the EsaI/EsaR library created for model fitting (part 1). The log-transformed optical density at 600 nm (OD600) experimental data is given in blue and the fit of the Richards growth curve is given in orange. The parameters corresponding to the fitted growth curves are given in Supplementary Table S1.

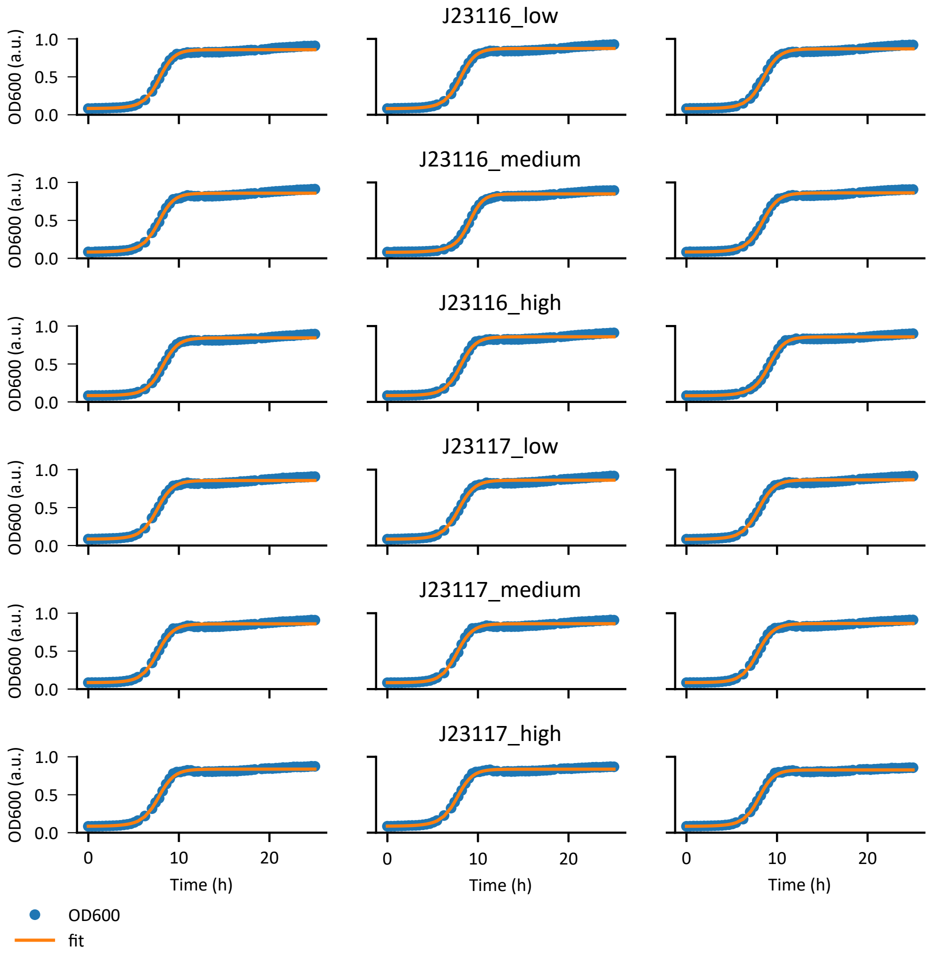

Figure S4: Growth curves of each of the three biological replicates of the fifteen strains of the EsaI/EsaR library created for model fitting (part 2). The log-transformed optical density at 600 nm (OD600) experimental data is given in blue and the fit of the Richards growth curve is given in orange. The parameters corresponding to the fitted growth curves are given in Supplementary Table S1.

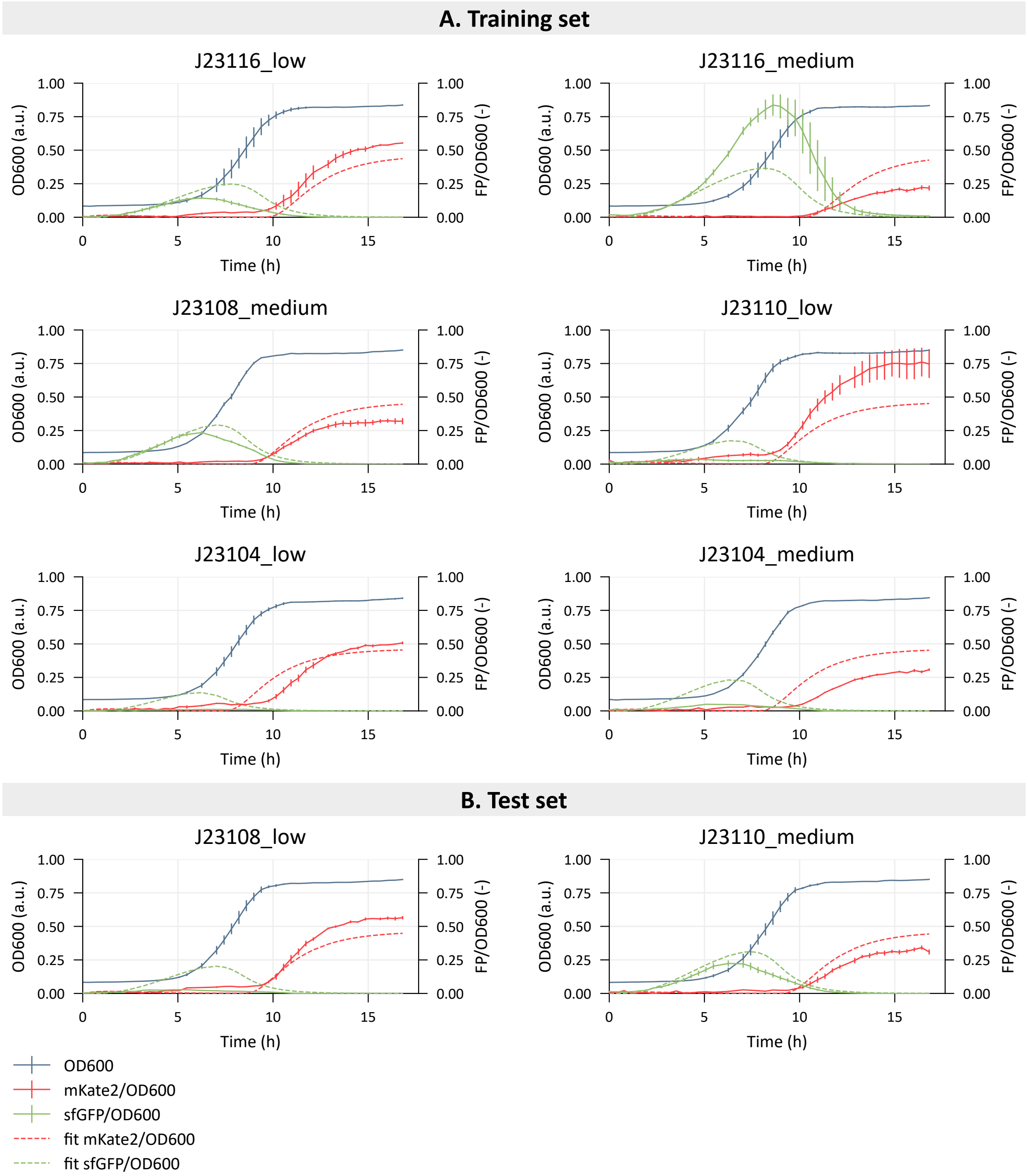

Figure S5: Model fit to the EsaI/EsaR library, strains with promoter Bba_J23104 included. The model was fit to the strains in the training set (**A.**) and applied to the strains in the test set (**B.**). The dashed lines depict the prediction made by the model. Fluorescent values are normalized for cell growth determined by optical density at 600 nm (OD600). Error bars represent the standard error for three biological replicates. FP = fluorescent protein, referring to either mKate2 or sfGFP.

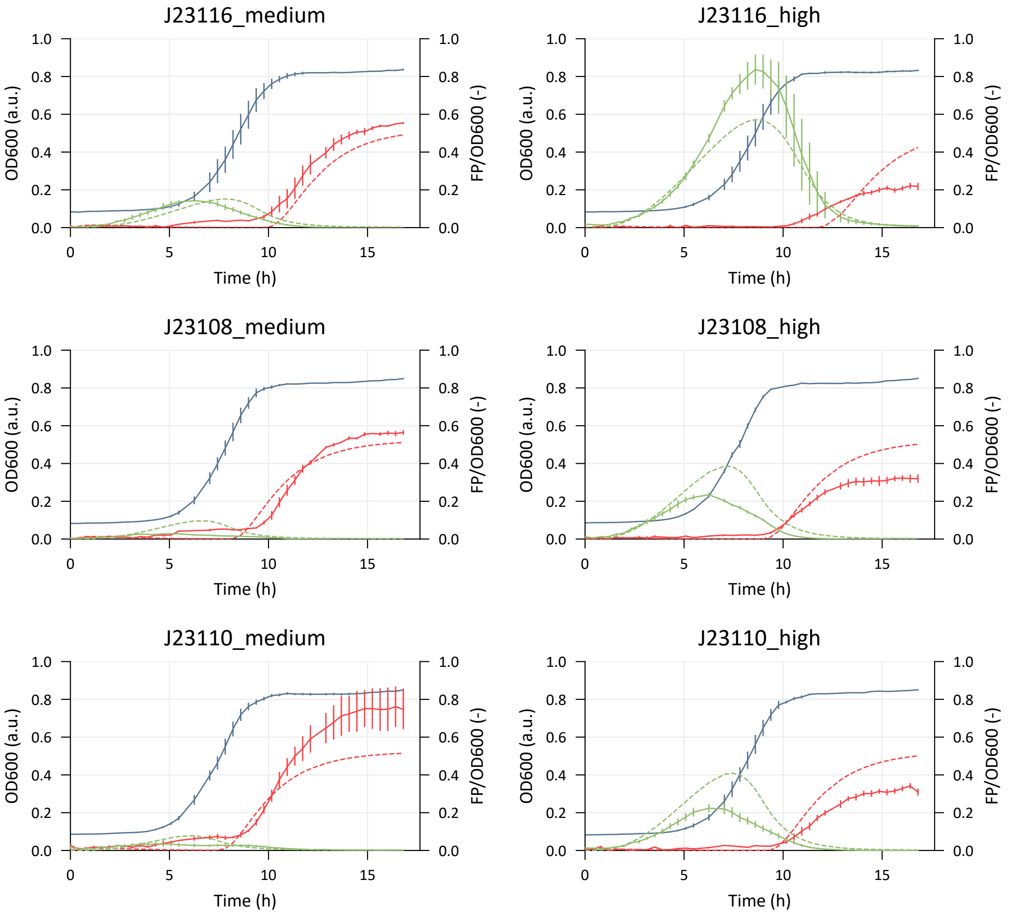

Figure S6: Fit of the model with high parameter correlations to the six strains of the EsaI/EsaR library. The dashed lines depict the prediction made by the model. Fluorescent values are normalized for cell growth determined by optical density at 600 nm (OD600). Error bars represent the standard error for three biological replicates. FP = fluorescent protein, referring to either mKate2 or sfGFP.

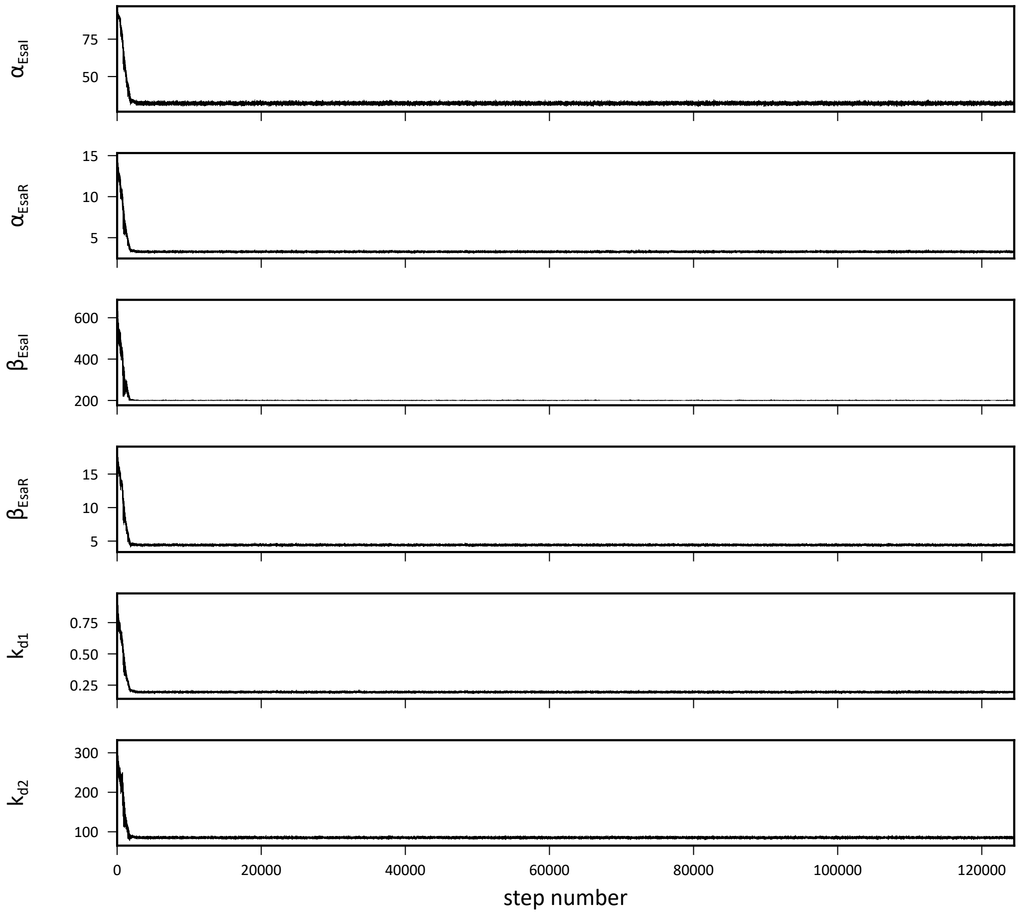

Figure S7: Traces of the 25 walkers of the Monte Carlo Markov Chain analysis for each of the parameters of the model with highly correlated parameters.

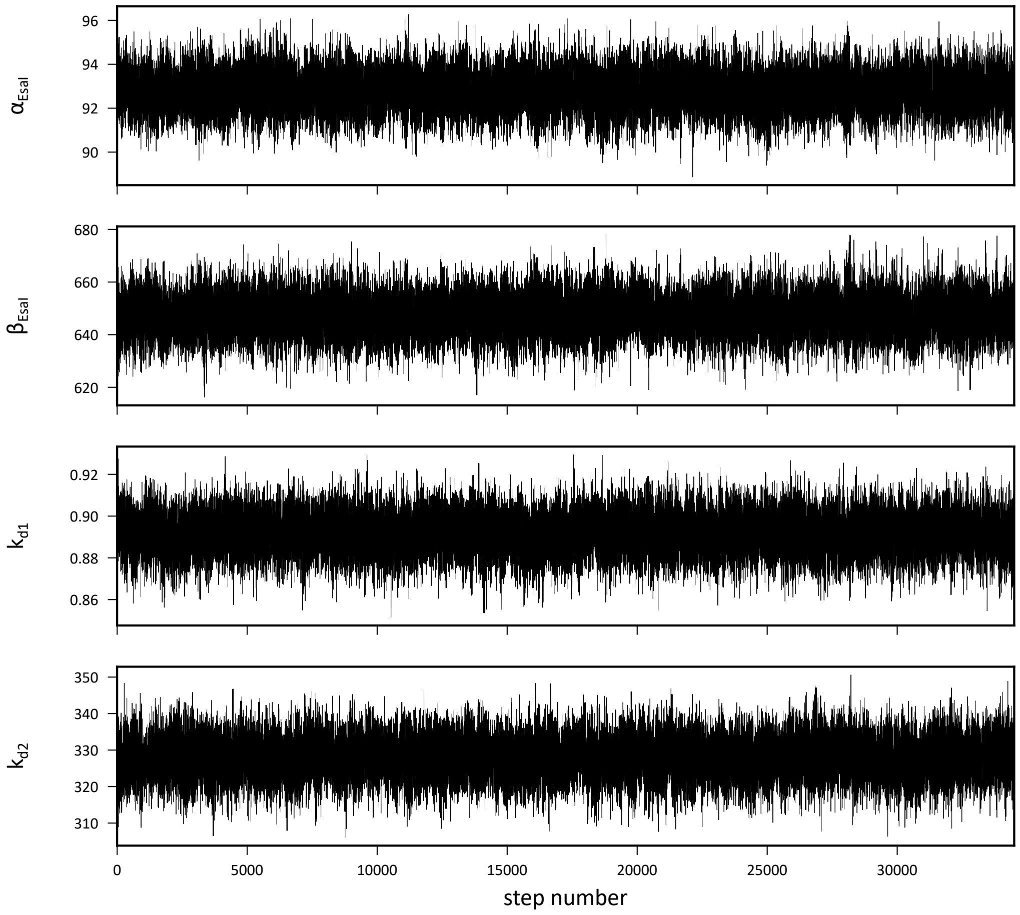

Figure S8: Traces of the 25 walkers of the Monte Carlo Markov Chain analysis for each of the parameters the model with reduced correlations between the parameters.

Table S1: Average growth curve parameters of the Richards growth curve fitted to all fifteen strains of the EsaI/EsaR library, with $OD600_0$ the optical density at 600 nm at timepoint 0, A the initial population density, μ_{max} the maximal specific growth rate, λ the lag time and ν a shape parameter.

Strain	$OD600_0$	A	μ_{max}	λ	ν
J23104_low	0.082996	2.356228	0.557687	4.93854	1.152714
J23104_medium	0.083657	2.332291	0.566096	4.82388	1.200786
J23104_high	0.081315	2.365061	0.573085	4.964603	1.56927
J23108_low	0.082988	2.324873	0.581977	5.223619	1.384739
J23108_medium	0.082665	2.352874	0.596934	4.777194	1.301487
J23108_high	0.085328	2.319001	0.578684	4.560795	1.237323
J23110_low	0.083332	2.349494	0.576107	4.540902	1.258389
J23110_medium	0.085996	2.305746	0.581192	4.369592	1.022652
J23110_high	0.082	2.364282	0.566679	5.000944	1.436458
J23116_low	0.080996	2.370093	0.577318	4.973286	1.661104
J23116_medium	0.08064	2.362861	0.569524	5.20053	1.901419
J23116_high	0.082328	2.338328	0.571699	5.324063	1.658477
J23117_low	0.082996	2.33956	0.566913	4.665598	1.499344
J23117_medium	0.084665	2.320368	0.584386	4.696271	1.329948
J23117_high	0.084332	2.291097	0.559483	4.720177	1.67654

Table S2: Results of the six estimated parameters of the model fitted to the final six strains. The estimated standard and relative error are also provided. The initial value, the minimal value (Min) and maximal value (Max) were used as input during the parameter estimation.

Name	Value	Standard error	Relative error	Initial value	Min	Max
α_{EsaR}	15.18183	0.92035418	-6.06%	0	0	800
α_{EsaI}	92.45641	8.59868541	-9.30%	0	0	600
β_{EsaI}	642.566	57.0468926	-8.88%	1200	200	1200
β_{EsaR}	18.10534	1.48473226	-8.20%	150	1	1000
k_{d1}	1	1.58E-04	-0.02%	300	0.1	300
k_{d2}	299.9631	2.09423916	-0.70%	300	1	300

Table S3: Overview of the DNA sequence of the regulatory parts used in this research.

Part	DNA sequence
**Promoters**	
P22	TTGACATTTTGGAATAGATGTGATATAATGTGTACATAT
Bba_J23104	TTGACAGCTAGCTCAGTCCTAGGTATTGTGCTAGC
Bba_J23108	CTGACAGCTAGCTCAGTCCTAGGTATAATGCTAGC
Bba_J23110	TTTACGGCTAGCTCAGTCCTAGGTACAATGCTAGC
Bba_J23116	TTGACAGCTAGCTCAGTCCTAGGGACTATGCTAGC
Bba_J23117	TTGACAGCTAGCTCAGTCCTAGGGATTGTGCTAGC
PesaR/esaS	CCGCTAAACAACTGAAGCCATTGTAACCTCTGAATGATTCATTGTAAGTTACTCTTAAGTATCATCTTGCC<br>TGTACTATAGTGCAGGTTAAGTCCACGTTAAGTAAAAGAAGCAGC
**RBS**	
RBS_low	CGTCACACTACCCGCTAAGACCTGGCCCGCGCGTCAGCCGTCTCT
RBS_medium	CGTCACACTACCCGCTAACTGAAGCGGGTCATAGACCGCGTCTCT
RBS_high	CGTCACACTACCCGCTAGCCCCCCACAAATTTAAGATACGTCTCT
Bba_B030	TCTAGAGATTAAAGAGGAGAAATACTAG
Bba_B032	ATACTAGAGTCACACAGGAAAGTACTAG
RBS45	ACACGATCTTCGAAGGACGTACAT
**Terminators**	
TT3-rrnD1-T1	GGGAACTGCCAGACATCAAATAAAACAAAAGGCTCAGTCGGAAGACTGGGCCTTTTGTTTTATCTGTTG<br>TTTGTCGGTGAACACTCTCCC
TT8-T3 TE	CCCTCAAGAGAAAATGTAACCAACTCACTGGCTCACCTTCACGGGTGGGCCTTTCTTCGTTCCGGGCATT<br>AACCCTCACTAACAGGAGA
BioFab terminator_FAB391’	TCGGTCAGTTTCACCTGATTTACGTAAAAACCCGCTTCGGCGGGTTTTTGCTTTTGGAGGGGCAGAAAG<br>ATGAATGACTGTC

Table S4: Overview of the coding sequence of all genes used in this research.

Part	Coding sequence
EsaR	1 ATGTTCTCGT TCTTCCTGGA AAACCAGACC ATTACGGATA CGCTTCAGAC TTACATACAG AGAAAGTTAT
	71 CTCCGCTGGG TAGTCCGGAT TACGCTTACA CTGTTGTGAG CAAAAAAAAT CCTTCAAATG TTCTGATTAT
	141 TTCCAGTTAT CCTGACGAAT GGATTAGGTT ATACCGCGCT AACAACTTTC AGCTGACCGA TCCGGTTATT
	211 CTCACGGCCT TTAAACGCAC CTCGCCGTTT GCCTGGGATG AGAATATTAC GCTGATGTCC GACCTGCGGT
	281 TCACCAAAAT TTTCTCTTTA TCCAAGCAAT ACAACATCGT TAACGGCTTT ACCTATGTCC TGCATGACCA
	351 CATGAACAAC CTTGCTCTGT TGTCCGTGAT CATTAAAGGC AACGATCAGA CTGCGCTGGA GCAACGCCTT
	421 GCTGCCGAAC AGGGCACGAT GCAGATGCTG CTGATTGATT TTAACGAGCA GATGTACCGC CTGGCCGGTA
	491 CCGAAGGCGA GCGAGCCCCG GCGTTAAATC AGAGCGCGGA CAAAACGATA TTTTCCTCGC GTGAAAATGA
	561 GGTGTTGTAC TGGGCGAGTA TGGGCAAAAC CTATGCTGAG ATTGCCGCTA TTACGGGCAT TTCTGTGAGT
	631 ACCGTGAAGT TTCACATCAA GAATGTGGTC GTGAAACTGG GCGTCAGTAA CGCCCGACAG GCTATCAGAC
	701 TGGGTGTAGA ACTGGATCTT ATCAGACCGG CAGCATCAGC TGCAAGGTAG
EsaI	1 ATGCTTGAAC TGTTTGACGT CAGTTACGAA GAACTGCAAA CCACCCGTTC AGAAGAACTT TATAAACTTC
	71 GCAAGAAAAC ATTTAGCGAT CGTCTGGGAT GGGAAGTCAT TTGCAGTCAG GGAATGGAGT CCGATGAATT
	141 TGATGGGCCC GGTACACGTT ATATTCTGGG AATCTGCGAA GGACAATTAG TGTGCAGCGT ACGTTTTACC
	211 AGCCTCGATC GTCCCAACAT GATCACGCAC ACTTTTCAGC ACTGCTTCAG TGATGTCACC CTGCCCGCCT
	281 ATGGTACCGA ATCCAGCCGT TTTTTTGTCG ACAAAGCCCG CGCACGTGCG CTGTTAGGTG AGCACTACCC
	351 TATCAGCCAG GTCCTGTTTT TAGCGATGGT GAACTGGGCG CAAAATAATG CCTACGGCAA TATCTATACG
	421 ATTGTCAGCC GCGCGATGTT GAAAATTCTC ACTCGCTCTG GCTGGCAAAT CAAAGTCATT AAAGAGGCTT
	491 TCCTGACCGA AAAGGAACGT ATCTATTTGC TGACGCTGCC AGCAGGTCAG GATGACAAGC AGCAACTCGG
	561 TGGTGATGTG GTGTCACGTA CGGGCTGTCC GCCCGTCGCA GTCACTACCT GGCCGCTGAC GCTGCCGGTC
	631 TGA

Table S4: Continued overview of the coding sequences used in this research.

Part	Coding sequence
sfGFP(ASV)	1 ATGAGCAAGG GCGAAGAGCT TTTTACCGGT GTTGTGCCGA TTTTAGTAGA ACTGGACGGA GACGTGAACG
	71 GTCATAAGTT CTCTGTTCGT GGCGAAGGAG AGGGAGATGC CACCAATGGT AAGCTGACCC TGAAGTTCAT
	141 CTGTACCACC GGTAAGCTGC CCGTGCCTTG GCCGACGCTG GTCACAACGT TGACGTATGG CGTCCAATGC
	211 TTTTCACGCT ATCCAGATCA CATGAAACGC CACGACTTTT TTAAAAGCGC AATGCCTGAA GGTTATGTGC
	281 AGGAACGGAC TATTAGCTTC AAAGACGATG GGACGTATAA GACCCGCGCG GAAGTGAAAT TTGAAGGCGA
	351 TACCTTAGTT AACCGCATTG AATTAAAAGG TATCGATTTC AAAGAGGATG GGAATATCCT GGGGCACAAA
	421 TTGGAATACA ACTTTAATTC GCACAACGTA TACATTACAG CGGATAAACA GAAAAATGGC ATCAAAGCCA
	491 ACTTTAAAAT CCGTCATAAC GTAGAGGACG GTTCCGTGCA GCTGGCTGAT CATTACCAGC AGAATACTCC
	561 GATTGGCGAT GGCCCCGTTC TGCTCCCGGA TAATCATTAC CTGTCTACAC AAAGCGTTCT TAGTAAAGAC
	631 CCAAACGAGA AGCGTGACCA TATGGTCCTG TTGGAATTCG TCACGGCAGC GGGGATTACT CATGGCATGG
	701 ATGAACTCTA TAAGGCAGCA AACGACGAAA ACTACGCTGC ATCAGTTTAA
mKate2	1 ATGGTTAGCG AGCTGATCAA AGAAAACATG CACATGAAAC TGTATATGGA AGGCACCGTG AATAACCACC
	71 ACTTTAAATG TACCAGCGAA GGTGAAGGTA AACCGTATGA AGGCACCCAG ACCATGCGTA TTAAAGCAGT
	141 TGAAGGTGGT CCGCTGCCGT TTGCATTTGA TATTCTGGCA ACCAGCTTTA TGTATGGCAG CAAAACCTTT
	211 ATTAACCATA CCCAGGGTAT CCCGGATTTT TTCAAACAGA GCTTTCCGGA AGGTTTTACC TGGGAACGTG
	281 TTACCACCTA TGAAGATGGT GGTGTTCTGA CCGCAACCCA GGATACCAGT CTGCAGGATG GTTGTCTGAT
	351 TTATAATGTG AAAATTCGCG GTGTGAACTT TCCGAGCAAT GGTCCGGTTA TGCAGAAAAA AACCCTGGGT
	421 TGGGAAGCAA GCACCGAAAC CCTGTATCCG GCAGATGGTG GTCTGGAAGG TCGTGCAGAT ATGGCACTGA
	491 AACTGGTTGG TGGTGGTCAT CTGATTTGCA ATCTGAAAAC CACCTATCGT AGCAAAAAAC CGGCAAAAAA
	561 TCTGAAAATG CCTGGCGTGT ATTATGTTGA TCGTCGTCTG GAACGTATTA AAGAGGCAGA TAAAGAAACC
	631 TATGTGGAAC AGCATGAAGT TGCAGTTGCA CGTTATTGTG ATCTGCCGAG CAAACTGGGT CACCGCTGA

Table S5: Overview of the DNA sequence primers used in this research.

Primer	Sequence (5' →3')
EsaR_sfGFPoverhang_Rv	CATACCAGAACCACCACCAGAACCACCCCTTGCAGCTGATGCTGCCGGTCTGATAAG
BB_sfGFPoverhang_pMoBioS-M4_Rv	ACTCATGGCATGGATGAACTCTATAAGTAACAATAGTCTTTCAGGGCCGTATGCAC
EsaI_sfGFPOverhang_Rv	CATACCAGAACCACCACCAGAACCACCGACCGGCAGCGTCAGC
TT8_sfGFPOverhang_Fw	ACTCATGGCATGGATGAACTCTATAAGTAACCCTCAAGAGAAAATGTAACCAACTC
pT7_sfGFP_Rv	TTACTTATAGAGTTCATCCATGCCATGAGTAATCCCCGCTGCCGTG
sfGFP_linker_FP	GGTTCTGGTGGTGGTTCTGGTATGGGCAAGGGCGAAGAGCTTTTTACCG
EsaI_BB_Rv	CCTTGAGTGGTGATTGATTG
J23104_EsaI_Fw	CAATCAATCACCACTCAAGGTTGACAGCTAGCTCAGTCCTAGGTATTGTGCTAGCACAC-GATCTTCGAAGGACG
J23110_EsaI_Fw	CAATCAATCACCACTCAAGGTTTACGGCTAGCTCAGTCCTAGGTACAATGCTAGCACAC-GATCTTCGAAGGACG
J23117_EsaI_Fw	CAATCAATCACCACTCAAGGTTGACAGCTAGCTCAGTCCTAGGGATTGTGCTAGCACAC-GATCTTCGAAGGACG
J23116_overhang_Fw	TTGACAGCTAGCTCAGTCCTAGGGACTATGCTAGCACACGATCTTCGAAGGACG
J23116_Rv	AGCATAGTCCCTAGGACTGAGCTAGCTGTCAACCTTGAGTGGTGATTGATTGAGC
**Internal primers**	
BB1_Rv	CTCGGATGGAAGCCGGTCTTGTCG
BB2_Fw	CGACAAGACCGGCTTCCATCCGAG
internal_pMoBioS_Rv	CCGCTAGCCCATGGTTATC
pBBR1MCS2-Psyn-BB_Fw	TAACCATGGGCTAGCGGTTTG
